# Clarifying the Dual Role of *Staphylococcus* spp. in Cheese Production

**DOI:** 10.3390/foods14223823

**Published:** 2025-11-07

**Authors:** Alessandra Casagrande Ribeiro, Déborah Tavares Alves, Gabriela Zampieri Campos, Talita Gomes da Costa, Bernadette Dora Gombossy de Melo Franco, Felipe Alves de Almeida, Uelinton Manoel Pinto

**Affiliations:** 1 Laboratory of Food Microbiology, Food Research Center (FoRC), Department of Food and Experimental Nutrition, Faculty of Pharmaceutical Sciences, University of São Paulo (USP), Av. Prof. Lineu Prestes 580, B.14, Butantã, São Paulo 05508-000, SP, Brazil; cribeiro.alessandra@gmail.com (A.C.R.); gabriela.zampieri.campos@usp.br (G.Z.C.); tgomes@usp.br (T.G.d.C.); bfranco@usp.br (B.D.G.d.M.F.); 2 University of Mogi das Cruzes (UMC), Mogi das Cruzes Campus, Av. Dr. Cândido X. de Almeida e Souza 200, Centro Cívico, Mogi das Cruzes 08780-911, SP, Brazil; 3 Cândido Tostes Dairy Institute, Minas Gerais Agricultural Research Agency (ILCT EPAMIG), Rua Tenente Luiz de Freitas 116, Santa Terezinha, Juiz de Fora 36045-560, MG, Brazil; deborah.tavaresalves@gmail.com; 4 Laboratory of Industrial and Food Microbiology (LAMIND), Institute of Biotechnology Applied to Agriculture (BIOAGRO), Department of Microbiology, Federal University of Viçosa (UFV), Vila Matoso 205, Santo Antônio, Viçosa 36570-000, MG, Brazil; felipe.alves@ufv.br

**Keywords:** coagulase-negative staphylococci, coagulase-positive staphylococci, enterotoxin, food safety, *Staphylococcus aureus*

## Abstract

*Staphylococcus* spp. present a dual role in cheese production as some species are pathogenic, while others bring beneficial characteristics. Coagulase-positive staphylococci (CoPS), particularly *Staphylococcus aureus*, are of concern due to their ability to produce enterotoxins linked to foodborne outbreaks. These toxins, encoded by staphylococcal enterotoxin (SE) genes, cause gastroenteritis, especially vomiting. Many members of the genus harbor a plethora of virulence genes and are able to form biofilms. The prevalence of antibiotic-resistant strains, including methicillin-resistant *S. aureus* (MRSA), complicates control. In contrast, some members of the coagulase-negative staphylococci (CoNS) group, such as *Staphylococcus carnosus*, *Staphylococcus condimenti*, *Staphylococcus equorum*, *Staphylococcus piscifermentans*, *Staphylococcus succinus*, and *Staphylococcus xylosus*, contribute to ripening, influencing flavor and texture. Some are even considered safe and studied for their ability to inhibit pathogens. Expression of enterotoxin genes in *Staphylococcus*, particularly *S. aureus*, is influenced by environmental factors and can be regulated by different mechanisms including quorum sensing. Understanding gene expression in conditions found during cheese production and ripening can help in formulating effective interventions. Risks posed by enterotoxin-producing *Staphylococcus* in cheese are evident, with numerous outbreaks reported worldwide. Moreover, several species present risks to both animal and human health. Effective control measures include adherence to microbiological criteria in foods, animal health monitoring, good manufacturing practices (GMP), temperature control, proper ripening conditions and hygiene. This review compiles and discusses existing knowledge on CoPS and CoNS in cheeses, providing a framework for evaluating their risks and benefits and guiding future studies in cheese microbiology.

## 1. Introduction

The genus *Staphylococcus* is composed of Gram-positive, spherical bacteria that characteristically divide in more than one plane, forming arrangements resembling clusters of grapes. These bacteria have a diameter between 0.5 and 1.5 μm, are non-motile and non-sporogenic, catalase-positive, and can be aerobic, facultative anaerobes or strictly anaerobic, such as *Staphylococcus aureus* subsp. *anaerobius* [1,2,3]. This group is susceptible to lysis by lysostaphin and resistant to lysis by lysozyme [4]. Acting either as commensals or opportunistic pathogens, they are taxonomically classified under the Kingdom Bacteria, Phylum Bacillota, Class Bacilli, Order Bacillales, and Family *Staphylococcaceae* [5].

The genus *Staphylococcus* is composed of 71 species and 14 subspecies, some of which are common inhabitants of the skin and respiratory tract of humans and warm-blooded animals, with few species that can be found in soil and aquatic environments [6,7]. In general, *Staphylococcus* spp. are present in most diverse environments and are considered symbionts [8]. According to Foster [9], *Staphylococcus* spp. are among the most resistant non-sporulating microorganisms: they can withstand high concentrations of salt, desiccation, heat, and are more tolerant to common disinfectants than most bacteria.

Species in the *Staphylococcus* genus are classified as coagulase-positive staphylococci (CoPS) or coagulase-negative staphylococci (CoNS) according to their ability to produce coagulase [4]. However, different strains from some species, such as *Staphylococcus hyicus*, present variation in coagulase production, being considered coagulase-variable staphylococci (CoVS) [10]. Most CoPS species are recognized as pathogenic, although some strains may asymptomatically colonize healthy individuals, while CoNS are primarily saprophytic or associated with opportunistic infections [4]. Coagulase is the main virulence factor of CoPS, functioning as a critical mechanism of defense by inducing fibrin deposition around the cells, protecting them in the infected area. Moreover, there is a correlation between CoPS and enterotoxin production, further enhancing their pathogenicity [11,12].

The first time *Staphylococcus* was associated with foodborne illness dates back to as early as 1884 when spherical organisms in cheese caused a large food-poisoning outbreak in the United States. Other outbreaks attributed to the consumption of staphylococcal contaminated foods occurred in France in 1894, in the United States in 1907, and in the Philippines in 1914. In 1930, Gail Dack and his colleagues at the University of Chicago were able to demonstrate that the cause of a food poisoning that occurred from the consumption of a contaminated Christmas sponge cake with cream filling was due to a toxin produced by the isolated staphylococci [13].

*Staphylococcus* spp. comprise a diverse genus that includes both pathogenic and beneficial species widely distributed in food-related environments. The cheese matrix, in particular, represents a complex ecological niche where this dual role becomes especially evident. Thus, this review explores the dual role of *Staphylococcus* spp. in cheese production, highlighting both the beneficial contributions of CoNS to cheese ripening and the risks posed by CoNS and CoPS, particularly *S. aureus*, due to their potential to produce enterotoxins and to form biofilms. The review also examines the regulatory mechanisms of enterotoxin gene expression, including quorum sensing, and discusses effective control measures to minimize the risks associated with enterotoxin-producing strains in cheese. Ultimately, this work shows that not all *Staphylococcus* spp. are detrimental in the production of cheese products.

## 2. Coagulase-Positive Staphylococci (CoPS)

The *coa* gene, responsible for encoding coagulase production, plays a pivotal role in the virulence of CoPS, especially in *S. aureus*, by facilitating the conversion of fibrinogen into fibrin, which aids in clot formation and immune evasion. Genetic analyses have revealed considerable variability in the *coa* gene, indicating the adaptability of CoPS in diverse environments [14]. These genetic variations can occur both chromosomally and through horizontal gene transfer via plasmids, contributing to the spread of virulent and antimicrobial-resistant strains. Notably, livestock-associated methicillin-resistant *S. aureus* (LA-MRSA) strains have been shown to harbor novel recombinant staphylocoagulase types, highlighting the significant role of horizontal gene transfer in the evolution and dissemination of these strains [15]. Phylogenetic analyses of CoPS isolates demonstrate distinct clusters based on *coa* types, which are often associated with specific infection sites and geographic origins [16].

The CoPS species (*n* = 9) are shown in Table 1. All other species are CoNS (*n* = 58), except for a few species that are CoVS (*n* = 4) [7,10,17,18,19]. Five species that belonged to the genus *Staphylococcus* were reclassified to the genus *Mammaliicoccus*: *M. fleurettii*, *M. lentus*, *M. sciuri*, *M. stepanovicii*, and *M. vitulinus* [19].

### 2.1. Staphylococcus aureus

*S. aureus* is a highly adaptable pathogen with a remarkable ability to thrive in diverse environments, contributing to its broad spectrum of infections. It can grow across a wide range of conditions, including temperatures from 7 to 48.5 °C (optimal 30–37 °C), pH levels from 4.2 to 9.3 (optimal 7.0–7.5), and sodium chloride concentrations up to 15% [20,21]. This adaptability makes *S. aureus* particularly significant in food safety, especially in foods requiring extensive handling during processing.

The virulence of *S. aureus* is driven by a variety of mechanisms, including the production of toxins such as alpha-hemolysin and Panton-Valentine leukocidin (PVL), along with superantigens like toxic shock syndrome toxin-1 (TSST-1) and staphylococcal enterotoxins (SEs). These virulence factors can lead to severe conditions such as tissue necrosis, vascular thrombosis, and bacteremia [22]. In the context of foodborne illness, staphylococcal food poisoning (SFP) is specifically attributed to the production of SEs, which are the primary virulence factor responsible for the gastrointestinal symptoms [23].

*S. aureus* is also noted for its role in hematogenous metastasis, biofilm formation, and the persistence of chronic infections, contributing to its ability to evade the immune system and resist treatment with antibiotics [24]. The combination of toxin production, invasiveness, and antibiotic resistance enables *S. aureus* to cause a wide range of symptoms, from superficial skin infections to more severe illnesses like toxic shock syndrome (TSS-like).

Strains of *S. aureus* can be classified into two groups based on their resistance to oxacillin/methicillin: methicillin-susceptible *S. aureus* (MSSA) and methicillin-resistant *S. aureus* (MRSA). MRSA strains emerged soon after the introduction of semisynthetic penicillins [25] and these strains are resistant to nearly all beta-lactam antibiotics, with the exception of ceftaroline and ceftobiprole [26]. Its clinical relevance lies in its association with poor prognoses and increased healthcare demands, as patients with MRSA infections often face longer hospitalizations, more extensive diagnostics, and higher mortality rates [27]. While some data suggests a decline in the proportion of MRSA isolates, it remains a critical pathogen in the European Union/European Economic Area (EU/EEA), especially in Southern and Eastern European countries, where resistance levels remain high [28,29,30].

The presence of *S. aureus* in many environments is a significant concern due to its potential to cause severe infections and its increasing resistance to antibiotics. Thus, effective monitoring and control strategies are essential to reduce the risk posed by this pathogen in both clinical and food safety contexts.

### 2.2. Other Coagulase-Positive Staphylococci (CoPS)

Other CoPS, such as *S. intermedius* and *S. coagulans*, as well as *S. hyicus*, a CoVS, also pose significant health risks. These species, alongside *S. aureus*, can produce enterotoxins and coagulase, contributing to foodborne illnesses and animal infections.

*S. aureus* subsp. *aureus* is the most extensively studied subspecies and is a common cause of foodborne diseases through the production of heat-stable enterotoxins, leading to SFP [16,20].

In veterinary medicine, *S. hyicus* is a significant pathogen in swine, causing exudative epidermitis, also known as “greasy pig disease”. While primarily of concern in veterinary contexts, human infections, though rare, have been documented [31].

*S. intermedius* is commonly associated with animals such as dogs, and rarely as the cause of SFP in humans. However, it was implicated in an outbreak in 1991, when more than 265 people in the western United States became ill after consuming food contaminated with *S. intermedius* [32,33]. Its close relatives, *S. pseudintermedius* and *S. delphini*, are known to colonize various animal species, with increasing antibiotic resistance adding to their public health concern [34,35]. *S. pseudintermedius*, primarily associated with canine and feline infections like pyoderma and otitis externa, has gained attention due to the emergence of methicillin-resistant strains (MRSP), presenting a challenge similar to that of MRSA in humans. The rise of MRSP underscores the need for improved infection control measures in veterinary healthcare [35,36].

Other CoPS, such as *S. lutrae*, have been predominantly isolated from wildlife, like otters [37], with no evidence of human infection. Meanwhile, *S. coagulans* has been linked to infections in dogs and occasional human cases, particularly in immunocompromised individuals [38]. While more commonly associated with skin infections in companion animals, *S. coagulans* can also pose a food safety risk due to its presence in animal hosts.

*S. delphini* and *S. argenteus* have been isolated from both animals and humans. *S. delphini* is mainly found in dolphins and horses, while *S. argenteus*, closely related to *S. aureus*, has emerged as a significant human pathogen [39]. Finally, *S. schweitzeri*, primarily isolated from primates, shares genetic similarities with *S. aureus* [40], raising concerns about its zoonotic potential.

## 3. Coagulase-Negative Staphylococci (CoNS)

Most research on antibiotic resistance of staphylococci isolated from food has focused on the species *S. aureus*, while less attention has been paid to the CoNS group [41]. For many years, CoNS were considered non-pathogenic and were usually identified only at the genus level. Their role in food can be considered dual in nature, as some species bring beneficial characteristics to food, while others may be pathogenic.

CoNS belong to the saprophytic microbiota of the skin and mucous membranes of warm-blooded animals and humans, but are also found in foods such as meat, cheese and milk [42]. Their incidence in food is much higher than that of CoPS and, generally, most species are commensals; however, in other circumstances, some can act as pathogens [42,43]. The CoNS group has 58 species, some of which have generally recognized as safe (GRAS) status and are considered positive microbiota as they are responsible for the organoleptic characteristics of the final products. Some CoNS can even be used as starter culture in the production of cheeses, sausages, and fermented meats, due to their aromatic and pigmenting capacity [44].

In dairy products, especially on the surface of various types of cheese, CoNS are frequently found, either as useful species to determine flavor or develop organoleptic properties or as contaminating species. Their presence may not be an immediate hazard to public health, but they can become a risk factor [45]. Some CoNS species may play a beneficial role in producing certain fermented foods. However, safety concerns arise due to identified risk factors associated with some strains, as well as reports of nosocomial and urinary tract infections linked to *S. epidermidis* and *S. saprophyticus*, which are CoNS species commonly found in fermented foods [46,47,48].

In processed foods, CoNS may be indicative of hygiene failures in handling [47], and in foods derived from raw milk, in particular, they are of great importance, since *Staphylococcus* spp. are the most common causes of mastitis [49]. Risk factors that have also been identified correspond to virulence, in particular, the production of enterotoxins, antibiotic resistance, and the ability to adhere and form biofilms [48,50,51,52].

SFP origin is among the most common foodborne diseases and, contrary to what was previously thought, can be associated with both CoPS and CoNS strains [53]. This generates increasing interest in CoNS strains, since they have been associated with infections in humans, and in the induction of SFP, due to the ability of some strains to produce enterotoxins [54,55]. Food processing does not eliminate these toxins, which, unlike bacteria, have greater resistance to high temperatures, a wide pH range, and proteolytic enzymes [56]. The toxins are also resistant to drying or freezing and are insensitive to enzymatic digestion in the human gastrointestinal tract [57].

For a long time, the production of cytolytic toxins was attributed exclusively to *S. aureus*, but toxigenic factors or corresponding genes have also been detected in *S. epidermidis* and other CoNS species [58]. Exfoliative toxins (ETs), including ExhA, ExhB, ExhC, and ExhD, have been identified in some strains of *S. hyicus*. These toxins likely cause exudative epidermitis in pigs, a skin lesion that has several features in common with staphylococcal scalded skin syndrome (SSSS) in humans and share sequence similarities with the ETs of *S. aureus*: ETA, ETB, and ETD. Furthermore, TSST-1-associated enterotoxins and ETs were identified in a CoNS collection, following detection of hemolytic activities during a comprehensive immunoblot analysis, where a significant proportion of the tested strains produced the toxins [59].

SEs are toxins that cause vomiting after reaching the gastrointestinal tract, but other toxins, called staphylococcal enterotoxin-like proteins (SEls) and that lack the ability to induce vomiting, can also be produced [60]. More than 24 different serological types of SE have been identified in strains from different foodborne outbreaks, clinical cases or isolated from animals. The first five SE genes identified, which code for SEA, SEB, SEC, SED, and SEE, known as classical enterotoxins, are frequently linked to foodborne outbreaks due to their ability to induce vomiting in humans. SEls are also considered a threat to humans, since they have been identified in cases of SFP outbreaks even without the presence of SEs. In addition, the new SE genes and the TSST-1, which belongs to a family of SE-associated toxins, are capable of stimulating large populations of T cells [61] (Table 2).

A study used polymerase chain reaction (PCR) and/or DNA microarrays to determine whether SE genes, which cause SFP and are typically associated with *S. aureus*, were also present in other *Staphylococcus* species isolated from food. The results revealed that the occurrence of SE genes in these isolates from products such as Naples-style salami, raw buffalo milk, and natural whey starter cultures (used for mozzarella cheese production) is very rare [65]. Unlike that, Nunes et al. [66] isolated CoNS from Minas Frescal cheese sold in southeastern Brazil, and all strains presented multiple SE genes, with *sea* and *seb* being the most frequently detected genes (90 and 70%, respectively), followed by *sec*/*see*, *seh*/*sei*, and *sed* with intermediate incidence (60, 50, and 40%, respectively). The lowest incidence was observed for *seg*/*selk*/*selq*/*selr* and *selu* (20 and 10%, respectively). Notably, the most frequent species were *S. saprophyticus* (40%), *S. xylosus* (30%), *M. sciuri* (20%, former name *S. sciuri*), and *S. piscifermentans* (10%). This divergence in results suggests that variations in the cheese matrix (fresh versus ripened/fermented products) and physicochemical parameters (pH, salt concentration, water activity—Aw, and presence of nitrite/nitrate) impose strong selective pressures on microorganisms, favoring or inhibiting the survival of toxigenic strains.

Additionally, Andrade et al. [67] observed that among the CoNS and CoPS species with enterotoxigenic potential, the *seg* and *seh* genes occurred in the species *S. cohnii* subsp. *cohnii*, *S. chromogenes*, *S. epidermidis*, *S. hominis*, *S. hyicus*, *S. lugdunensis*, *S. saprophyticus*, *S. ureilyticus*, and *S. xylosus*, with *seg* gene being the most predominant.

Chajecka-Wierzchowska et al. [68] evaluated 118 CoNS isolates from ready-to-eat foods, including sushi, salads, natural juices, hamburgers, beef tartare, and salmon tartare, obtained from bars and restaurants in Poland and observed that 72% were positive for at least one gene encoding for enterotoxin, while 28% were negative for the genes tested. The study also examined the presence of exfoliative genes (*eta*, *etd*), as well as the *tsst-1* gene. The presence of the *tsst-1* gene encoding TSST-1 was confirmed in 31.4% of CoNS strains belonging to the following species: *S. simulans* (n = 8), *S. carnosus* (n = 6), *S. epidermidis* (n = 3), *S. warneri* (n = 3), *S. xylosus* (n = 3), *S. saprophyticus* (n = 2), *S. pasteuri* (n = 1), *S. petrasii* (n = 1), and *S. piscifermentas* (n = 1). Although some isolates carried toxin-encoding genes, none exhibited phenotypic toxin expression under laboratory conditions. Toxin production is influenced by multiple factors, and the absence of phenotypic expression of these genes may result from genetic mutations or from the lack of regulatory elements required for operon activation. Additionally, toxin synthesis can occur only under specific environmental conditions, such as optimal temperature, pH, nutrient availability, and other influencing factors.

Other species of the *Staphylococcus*, especially the CoNS, have significant roles as infectious agents for human or animal hosts, revealing a more restricted repertoire of virulence factors when compared to *S. aureus*. They act as infectious agents, with moderately pathogenic species typically causing subtler infections characterized by a subacute or chronic clinical course. These infections rarely present with fulminant signs and are seldom fatal [69]. The most notable representative of this group is *S. epidermidis*.

Found widely on human skin, wounds or in surgical sites, which may be the factors for the entry of this microorganism into the host’s bloodstream, *S. epidermidis* has been highly related to hospital infections. Like *S. aureus*, *S. epidermidis* strains are highly resistant to antibiotics. The *S. epidermidis* species comprises a group of pathogens characterized by pronounced genomic diversity and when detected in clinical samples, clinicians face the challenge of determining whether they represent a true infection or just colonization/contamination [70]. With great clinical impact, this species has become the most important model microorganism for the study of healthcare-associated infections linked to inserted or implanted medical devices [71].

In addition to *S. epidermidis*, species such as *S. saprophyticus*, *S. haemolyticus*, and *S. lugdunensis* are occasionally observed as infectious agents of humans and animals, especially in patients with compromised immune systems [70,72].

### Generally Recognized as Safe (GRAS) Status

In order for a microorganism to be used in the preparation and composition of a food, it must have GRAS status. Many non-pathogenic *Staphylococcus* species are used in the food industry because they confer unique characteristics to products. Among them, some CoNS species stand out, such as *S. carnosus*, *S. condimenti*, *S. equorum*, *S. piscifermentans*, *S. succinus*, and *S. xylosus.* Among these, *S. carnosus*, *S. equorum*, *S. succinus*, and *S. xylosus* are used as starter cultures for the production of cheeses and fermented meat products [49].

CoNS play a significant role in defining color and developing organoleptic characteristics, which vary according to their proteolytic and lipolytic abilities [73,74]. This bacterial group can also contribute to the sensory qualities by producing diverse aroma profiles through carbohydrate and amino acid catabolism, ester formation, interactions with fatty acids, and their protease and lipase activities. Based on these distinctive properties, CoNS can be selected for use as starter cultures for fermentation [75].

*S. carnosus* has been used as a starter culture in the food industry since the 1950s. Its genome sequence has been determined and has provided the means for comparative studies of pathogenic and nonpathogenic staphylococci, and it has also been used as a cloning host to study the function of specific staphylococcal genes, given its food-grade status [76,77].

*S. xylosus*, belonging to the novobiocin-resistant CoNS species group, is commonly isolated from human and animal skin. This species is used as a starter culture in the fermentation of meats and cheeses because it enhances product safety and extends shelf life, while also contributing desirable sensory attributes. In cheeses, it plays a key role in the development of texture, flavor, and aroma through its proteolytic and lipolytic activities, and it contributes to the orange coloration of the surface via nitrite and nitrate reductase and catalase activities [78].

When evaluating strains of *S. equorum* isolated from cured cheese, the strain WS 2733 demonstrated the secretion of the macrocyclic peptide antibiotic micrococcin P(1), which exhibits antilisterial activity. This property was explored in cheese fermentation as a means to control the contamination by *Listeria monocytogenes* [79]. Deetae et al. [80] evaluated the production of volatile aromatic compounds by CoNS bacterial strains, isolated from different French cheeses, observing that *S. equorum* produced volatile compounds such as 3-methyl-3-buten-1-ol and 4-methyl-2-pentanone, responsible for conferring the fruity and sweet characteristics to the cheese. The aromatic compounds present in cheese mainly originate from three metabolic pathways: the catabolism of lactose and organic acids, protein catabolism, and lipid catabolism. These pathways can be activated by coagulation enzymes, endogenous milk enzymes, and microbial enzymes acting during manufacture and ripening. The main enzymes produced by microorganisms involved in protein catabolism include deaminases, decarboxylases, transaminases, lyases, and dehydratases. These enzymes are responsible for generating amines, aldehydes, alcohols, acids, and sulfur compounds. The lipid catabolism leads to the formation of esters, methyl ketones, and secondary alcohols, with esters being primarily responsible for the fruity flavor notes.

Other CoNS species also produce compounds of interest such as diacetyl and acetoin, observed in *S. succinus* and *S. xylosus*, when isolated from fermented sausage. Regarding the enzymatic activity of CoNS, some have amino acid converting enzymes and specific peptide uptake mechanisms to produce volatile aroma compounds. These mechanisms and metabolic pathways involved in the production of compounds of interest will vary depending on the CoNS species [81,82]. Thus, the addition of CoNS as starter cultures in the fermentation of cheeses and meats proves to be a safe alternative capable of conferring desired sensory characteristics.

Lee et al. [83] evaluated the genetic potential of *S. equorum* KS1039 as a starter culture in the fermentation of high-salt foods and observed that this strain contains genes for the biosynthesis of all amino acids except asparagine. Moreover, this strain harbors genes for the biosynthesis of branched-chain fatty acids such as α-acetolactate synthase (*als*), α-acetolactate decarboxylase (*adc*), and 2,3-butanediol dehydrogenase (*bdh*). This pathway enables the regeneration of NAD^+^ from pyruvate and is linked to the production of aroma compounds such as diacetyl, acetoin, and 2,3-butanediol, which contribute buttery or creamy notes to fermented foods. In addition, *S. equorum* KS1039 possesses a diverse set of enzymes for amino acid catabolism and can produce methyl-branched ketones from branched-chain amino acids such as leucine, isoleucine, and valine, compounds that are important contributors to the volatile aroma profile of fermented products.

Irlinger et al. [84] evaluated the genome sequence of *S. equorum* Mu2 from a French ripened cheese and observed that the strain did not possess any of the virulence factors found in *S. aureus.* Genomic evaluation of *S. succinus* 14BME20, isolated from fermented soybeans, confirmed that it did not contain any of the known *S. aureus* virulence factor-encoding genes, but it did contain strain-specific genes for lipid degradation, which may contribute to the production of volatile compounds [85].

## 4. Virulence Factors

Among the various *Staphylococcus* species, *S. aureus* stands out as both a tolerated commensal and a potent pathogen, widely colonizing several animals, the human skin and mucous membranes, as well as being present in food. In addition to the production of toxins, its pathogenicity is attributed to a diverse arsenal of virulence factors that facilitate adhesion to host tissues, biofilm formation, immune system evasion, and survival under nutrient-limited conditions. Additionally, the ability to acquire antibiotic resistance further enhances its clinical relevance. However, due to its genomic plasticity, not all *S. aureus* strains share the same genetic composition, leading to significant variability in virulence and pathogenic potential among subpopulations. The expression of these factors is influenced by environmental conditions and the host’s immune response, determining the strain’s capacity to cause infections ranging from mild skin lesions to severe systemic diseases, as well as its ability to form biofilms and produce enterotoxins in food. Given its broad impact on human and animal health, *S. aureus* is considered the primary reference for comparing pathogenic *Staphylococcus* species [78,86].

Just as *Staphylococcus* spp. play a dual role in cheese production, biofilm formation by CoPS and CoNS also exhibits this duality, depending on the characteristics of the producing strain. Strains carrying antibiotic resistance genes and enterotoxin expression genes are particularly concerning in cheese.

### 4.1. Biofilm Formation

Biofilm formation by CoPS and CoNS plays a significant role in the persistence of these bacteria on both biological materials and inert surfaces, such as cheese, equipment, and utensils used in production. Biofilm-associated cells are highly adherent and exhibit reduced susceptibility to desiccation, heat, detergents, biocides, and other antimicrobial agents [87,88,89]. In dairy processing environments, these biofilms are difficult to eliminate through conventional sanitation procedures, representing persistent sources of cross-contamination and posing challenges to microbiological control. When formed by enterotoxigenic strains and/or those carrying antibiotic resistance genes, such biofilms represent a significant threat to food safety, as the microorganisms can withstand adverse processing conditions and remain viable in the final product. Conversely, biofilm formation by GRAS-status CoPS and CoNS may have beneficial effects, particularly when these strains contribute to cheese ripening, promote the development of characteristic flavor and texture, and exhibit antagonistic activity against foodborne pathogens. Thus, biofilm formation by CoPS and CoNS can be seen as both a threat and an ally in cheese production, depending on the microbiological characteristics of the strains involved.

Several studies highlight the ability of different *Staphylococcus* spp. isolates from cheese to form biofilms. Friedriczewski et al. [90] tested 20 *S. aureus* isolates from buffalo mozzarella cheese, and observed that 10% were strong biofilm formers, 35% moderate formers, 50% weak formers, and 5% were non-biofilm formers. Souza et al. [91] analyzed 11 *S. aureus* isolates obtained from Minas Frescal and Porungo cheeses, and 55% of them were strong biofilm formers. Meanwhile, Pineda et al. [92] evaluated the capability of 54 genetically pulsotypes of *S. aureus* isolates from raw milk artisanal cheeses from Canastra (Brazil), observing that none of them was strong biofilm former, while 24% were moderate and 16.7% did not form biofilm. Moreover, Carvalho et al. [93] demonstrated that *S. aureus* ATCC 25923 is capable of growing and forming biofilms on an 18-micron low-density polyethylene (LDPE) package when stored at 5 °C in the presence of Minas Frescal cheese whey. Additionally, bacterial cells were able to detach from the packaging, increasing the microbial load on the product.

Fontes et al. [89] showed that 29.5% of the 227 CoNS isolated from soft cheeses in Brazil were able to form biofilm. Gajewska and Chajęcka-Wierzchowska [94] isolated 54 staphylococcal strains from cow’s milk samples, of which 42 were classified as CoNS, belonging to the following species, *S. capitis*, *S. chromogenes*, *S. haemolyticus*, *S. hominis*, *S. saprophyticus*, *S. sciuri* (reclassified as *M. sciuri*), *S. simulans, S. warneri*, and *S. xylosus*, while 12 were classified as *S. aureus*. All tested isolates exhibited the capacity for biofilm formation. Of these, 85.7 and 58.3% of the CoNS and *S. aureus* isolates were capable of forming strong biofilms, while 4.8 and 8.3% formed moderate biofilms, and 9.5 and 33.3% formed weak biofilms, respectively. Interestingly, Goetz et al. [95] evaluated the effect of CoNS isolates with a weak-biofilm phenotype on the biofilm formation of other CoNS and CoPS isolates from the mastitis pathogen culture collection. Four of the CoNS isolates with a weak-biofilm phenotype (*S. chromogenes* C and E, and *S. simulans* F and H) significantly reduced biofilm formation in approximately 80% of the staphylococcal species tested, including *S. aureus*. These four *S. chromogenes* and *S. simulans* isolates were also able to disperse pre-established biofilms, but did not inhibit the growth of isolates with a strong-biofilm phenotype. These results suggest that some CoNS isolates can negatively affect the ability of other staphylococcal isolates and species to form biofilms via a mechanism that does not involve growth inhibition.

Formation of bacterial biofilms is a complex process comprising four main stages: adhesion, aggregation, maturation, and dispersion [96,97]. During the initial adhesion stage, *S. aureus* planktonic cells utilize various factors and regulatory mechanisms, such as the expression of cell wall-anchored (CWA) proteins, adhesins, and extracellular DNA (eDNA), to attach to biotic and abiotic surfaces [98,99]. One of the primary mechanisms involved is the organization of microbial surface components recognizing adhesive matrix molecules (MSCRAMMs), including protein A (SpA), fibronectin-binding proteins (FnBPs, such as FnbA and FnbB), fibrinogen-binding proteins (Fib), clumping factors (ClfA and ClfB), serine-aspartate repeat family proteins (SdrC, SdrD, and SdrE), biofilm-associated protein (Bap), and *S. aureus* surface proteins (SasC and SasG) [100,101,102,103,104,105]. Souza et al. [91] reported that among the 20 *S. aureus* isolates from Minas Frescal and Porungo cheeses, 85% and 50% exhibited expression of the *fnbA* and *clfB* genes, respectively. Pineda et al. [92] evaluated the virulence potential of 54 *S. aureus* isolates from raw milk artisanal cheeses from Canastra (Brazil) and found that most isolates possessed MSCRAMM genes, including *fnbA* (33.3%), *fnbB* (33.3%), *fib* (81.5%), *clfA* (98.1%), *clfB* (98.1%), and *eno* (98.1%).

SasC promotes the formation of large cell aggregates, increases adhesion to polystyrene, and enhances biofilm formation in *S. aureus* and *S. carnosus* [106]. SasG and plasmin-sensitive protein (Pls) from *S. aureus* are homologous to accumulation-associated protein (Aap) in *S. epidermidis* and other CoNS. Aap and SasG appear as long fibrils on the bacterial cell surface [107,108] and play roles in host cell binding and biofilm formation [108,109,110], while Pls surface expression reduces *S. aureus* adhesion to fibronectin [111,112,113]. Aap has also been shown to mediate intercellular adhesion in polysaccharide intercellular adhesion (PIA)-negative *S. epidermidis* strains, leading to a proteinaceous extracellular biofilm matrix [109]. Artini et al. [114] demonstrated that all 106 CoNS isolates from the surface of the Italian cheeses, including Casera Valtellina, Scimudin, Gorgonzola, Taleggio, and Formaggio di Fossa, carried the *aap* gene (100%), whereas the *atlE* gene was detected in only seven strains (6.6%). The authors also reported that the *atlE* gene was absent in all non-biofilm-producing strains. A class of bifunctional proteins known as adhesins facilitate biofilm attachment to host tissues and surfaces. These include AtlA and Aaa in *S. aureus* [115] and AtlE and Aae in *S. epidermidis* [116]. Adhesins play essential roles at multiple stages of biofilm formation and adhesion [117]. Although numerous surface adhesins have been identified, *S. aureus* possesses a much larger repertoire of these proteins than *S. epidermidis*, which is limited to a few adhesive proteins. The ability to attach to host tissues or surfaces is a prerequisite for the subsequent formation of multilayered biofilms, stabilized by exopolysaccharides or proteinaceous intercellular material [75].

Gajewska and Chajęcka-Wierzchowska [94] isolated 42 CoNS and 12 *S. aureus* from cow’s milk. They then identified genetic determinants responsible for biofilm formation, such as the *bap* and *eno* genes. Additionally, among CoNS, they detected the *aap*, *bhp*, *fbe*, *embP*, and *atlE* genes. Most of the tested staphylococcal strains (90.7%) had at least one of the tested genes. Nearly half (47.6%) of the CoNS had the *eno* gene, while for *S. aureus*, the *eno* gene was found in 58.3% of isolates. The frequency of the *bap* gene occurrence was 23.8% in CoNS strains and 25% in *S. aureus*, respectively. The *fbe* gene was demonstrated in only three CoNS isolates. Among the CoNS, the presence of the *embP* (16.7%), *aap* (28.6%), and *atlE* (23.8%) genes was also demonstrated. Following the adhesion step, bacterial cells begin to divide and aggregate [118]. During the aggregation stage, bacteria regulate biofilm formation by sensing environmental signals that activate regulatory networks and intracellular signaling molecules, promoting bacterial proliferation and biofilm thickening [119]. The biofilm provides resistance against the human immune system and antibiotics [120], while bacterial cells lose direct contact with the host surface and rely on cell–cell and cell–extracellular polymeric substance (EPS) adhesion [121].

Among the EPS components in *S. aureus* biofilms, PIA is biosynthesized as a poly-N-acetylglucosamine (PNAG) polymer and is a key factor [122]. PIA has cationic properties and plays a crucial role in adhesion and aggregation [123]. In *S. aureus*, biofilm formation is controlled by PIA production through proteins encoded by the *icaADBC* operon. Mutant strains lacking PIA exhibit significantly reduced bacterial cell adhesion [124]. PIA-dependent biofilms are predominantly observed in MSSA strains [124,125]. PIA interacts with other small proteins, such as Bap (biofilm-associated protein) that promotes cell-to-cell aggregation during biofilm formation [126] and Aap that facilitates biofilm maturation [127]. Souza et al. [91] reported that 90% of the 20 *S. aureus* isolates obtained from Minas Frescal and Porungo cheese expressed the *icaD* gene. Also, Pineda et al. [92] found that *icaA* and *icaD* genes were present in 70.37% and 46.2% of 54 *S. aureus* isolates from raw milk artisanal cheeses from Canastra (Brazil), respectively.

Although the *ica* operon is considered essential as the genetic basis for PIA production in biofilm formation, biofilm development through *ica*-independent mechanisms has also been observed. Specifically, *ica*-negative mutants of *S. epidermidis* can still form biofilms, though these exhibit a proteinaceous rather than polysaccharide composition, as evidenced by their resistance to metaperiodate and their susceptibility to protease disruption [128]. However, *ica*-negative strains such as *S. epidermidis* ATCC 12228 have been reported to lack biofilm formation capabilities [129]. Gajewska and Chajęcka-Wierzchowska [94] showed that, among the 42 CoNS and 12 *S. aureus* isolates from cow’s milk, the *icaA* was detected only in CoNS strains (24.1%), while *icaD* was found in both CoNS strains (21.4%) and *S. aureus* (100%).

Environmental factors present in cheese can directly modulate the expression of biofilm-related genes in *Staphylococcus* spp., although strain-dependent variations may occur [130,131]. In vitro studies have demonstrated that the expression of the *icaADBC* operon in *Staphylococcus* can be induced by high salt, high glucose, or ethanol. These findings suggest that similar conditions, such as the high osmolarity or salinity on the surface of ripening cheeses, combined with nutrient-rich environments, may act as inducing signals for *icaADBC* transcription, promoting PIA synthesis and biofilm formation. Moreover, the availability of carbon sources such as lactose and glucose in cheese and whey can enhance the activity of regulators such as SarA and SigB, which positively control *icaADBC* expression. In addition to this mechanism, the *bap* gene is often overexpressed under stress conditions, including elevated sodium chloride (NaCl) levels or nutrient fluctuations, which resemble the physicochemical environment of cheese surfaces. Taken together, these observations indicate that the physicochemical properties of cheese, particularly osmotic stress and nutrient availability, may create favorable conditions for *Staphylococcus* to activate biofilm-related genes and persist during cheese ripening [130].

During the maturation stage, biofilms become highly organized, forming compact, three-dimensional mushroom- or tower-like structures [132]. Channels develop around microcolonies, facilitating nutrient transport to deeper biofilm layers [133]. Mature biofilms exhibit metabolic diversity, which enhances their ability to withstand environmental stressors [134]. The production of EPS promotes bacterial aggregation into microcolonies, which serve as the structural foundation of the biofilm [135]. As these microcolonies thicken, genetic or environmental cues may trigger biofilm dispersion [136].

Biofilm dispersion is a complex, multi-step process that includes the production of exoenzymes and surfactants capable of degrading the EPS matrix [136], as well as physiological adaptations that prepare cells for survival outside the biofilm [137]. Once dispersed, cells revert to the planktonic state, allowing them to colonize new sites and initiate a new biofilm formation cycle [138]. As the final stage of the biofilm life cycle, dispersion plays a crucial role in infection spread. During biofilm growth and development, surfactant-like phenol-soluble modulins (PSMs) contribute significantly to biofilm dispersion and transmission in *S. aureus*. These molecules disrupt non-covalent interactions within the biofilm matrix and facilitate the formation of nutrient transport channels [139,140]. PSMs exist both in soluble form and as amyloid fibers, which provide structural stability to the biofilm [141,142].

The structural and functional complexity of biofilms increases as cells divide and the matrix becomes denser, creating physiological heterogeneity within the biofilm. This heterogeneity is characterized by gradients of nutrients and oxygen [143]. Within a biofilm, bacterial cells can be categorized into four distinct metabolic states: (i) aerobic cells, located in the oxygen- and nutrient-rich outer layer; (ii) fermentative cells, found in the oxygen- and nutrient-poor inner layer; (iii) dormant cells, residing in the anoxic layer with slow growth and inactive metabolism; and (iv) dead cells [144,145,146]. Dormant cells exhibit decreased intracellular adenosine triphosphate (ATP) levels, rendering them less susceptible to antibiotics [147]. Additionally, gradients of viscosity and lipid composition within *S. aureus* biofilms contribute to biofilm dispersal by facilitating the detachment of loosely bound bacteria while preserving a stable core layer [148,149].

A comprehensive overview of biofilm formation and regulatory mechanisms in *S. aureus* can be found in Peng et al. [96] and Wu et al. [97], whereas for *S. epidermidis* and other CoNS, see Schilcher and Horswill [98] and França et al. [150].

### 4.2. Antibiotic Resistance

*Staphylococcus* develops antibiotic resistance through multiple mechanisms that vary depending on the class of antibiotic but commonly involve modification of the drug’s target, enzymatic inactivation, and biofilm formation. These resistance mechanisms can be acquired through genetic mutations or horizontal gene transfer mediated by plasmids, transposons, integrons, and bacteriophages. In the cheese production chain, the transmission of antibiotic resistance can occur through multiple routes. Raw milk is a primary source, as *Staphylococcus* spp. and other bacteria from the animal microbiota or the milking environment may already harbor resistance genes. The use of sublethal concentrations of disinfectants or antibiotics in dairy farms may exert selective pressure that favors resistant strains. Consequently, resistant *Staphylococcus* spp. or their resistance genes can persist throughout processing, reaching the final product and potentially entering the human microbiota upon consumption. In addition, during cheese production, cross-contamination may occur via equipment, surfaces, or handlers, facilitating the transfer of resistance determinants. Environmental conditions during ripening, including microbial interactions within the cheese matrix, can also enhance genetic exchange among bacterial populations [151,152,153,154,155,156,157,158].

Resistance to beta-lactams in MRSA strains and many methicillin-resistant coagulase-negative staphylococci (MRCNS) strains is associated with the presence of transferable genomic islands (GI) in the bacterial genome, known as staphylococcal chromosomal cassette mec (SCCmec). The *mecA* gene, carried by the SCCmec, encodes penicillin-binding protein 2a (PBP2a), a transpeptidase with low affinity for beta-lactams, thereby conferring resistance to methicillin [159,160,161]. From an evolutionary perspective, the *mecA* gene found in *S. aureus* likely originated from a group of bacteria previously classified as CoNS, now reclassified under the genus Mammaliicoccus [161,162]. Different SCCmec types may harbor the *mecA* gene or others, along with resistance determinants for other antibiotic classes, such as aminoglycosides, macrolides, lincosamides, streptogramins B, and tetracyclines (MLS-B) [159,161].

The emergence of MRSA and MRCNS strains has left only a few antibiotics effective for treating infections. Even the use of glycopeptides, so-called last-resort antibiotics, such as vancomycin, is at risk of becoming ineffective [163]. Intermediate-susceptible *S. aureus* to vancomycin (VISA) and glycopeptides (GISA), as well as vancomycin-resistant *S. aureus* (VRSA; vancomycin MIC ≥ 16 mg/L), have also been reported [164]. Changes in the cell wall and metabolic pathways can lead to intermediate resistance to vancomycin [163], while the acquisition of the *vanA* resistance determinant results in high-level resistance to vancomycin [165]. Glycopeptide resistance, encoded by the *vanA* operon, is more frequently expressed in *S. aureus* strains with mutations in the modification-restriction system, and the presence of the pSK41-like conjugative plasmid and/or the Tn1546 transposon, both of which enhance the frequency of *vanA* operon conjugation [164,166,167,168,169].

CoPS and CoNS have also developed resistance to other classes of antibiotics, including aminoglycoside, diaminopyrimidine, fusidane, lincosamide, macrolide, nucleoside, phenicol, phosphonic acid, quinolone, streptogramin, tetracycline, and trimethoprim [161,164,170,171]. Other anti-MRSA antimicrobials have been developed, including daptomycin, linezolid, telavancin, tigecycline, quinupristin/dalfopristin, cephalosporins, and ceftobiprole. However, some strains have already developed resistance mechanisms to these new drugs [161,164,172].

Table 3 provides an overview of various resistance genes associated with different antibiotic classes and their corresponding encoded proteins in *Staphylococcus* spp. As shown in Table 3, one single gene may confer resistance to multiple antibiotics. Mlynarczyk-Bonikowska et al. [164], Brdová et al. [161] and Alkuraythi et al. [171] published comprehensive overviews on antibiotic resistance and the molecular mechanisms of this resistance in *S. aureus* and other CoPS and CoNS.

Thus, *S. aureus*, throughout its evolution, has acquired resistance to nearly all antibiotics developed so far. The presence of populations exhibiting multiple antibiotic resistances, which are highly prevalent in the environment, is a serious concern as it compromises the effectiveness of treatments for staphylococcal infections [83]. Furthermore, their antimicrobial resistance determinants may also be transferable to other commensal or potentially pathogenic bacteria in foodstuff [52,173,174]. Similarly, CoNS have also acquired resistance to various antibiotics throughout their evolution and may be present in cheeses, contributing to the transfer of resistance genes [66,89,175]. It is noteworthy that Fontes et al. [89] found high counts of CoNS in Brazilian soft cheeses, ranging from 10^6^ to 10^7^ CFU/g.

Gajewska et al. [176] conducted a study in Poland in which they tested 180 *S. aureus* isolates collected from various stages of artisanal cheese production using unpasteurized milk. The study revealed notable levels of antimicrobial resistance among the isolates: penicillin (58.1%), tobramycin (34.4%), azithromycin (18.3%), clarithromycin (16.1%), erythromycin (22.6%), cefoxitin (12.9%), and oxacillin (9.7%). The *blaZ* gene, which encodes penicillin resistance, was the most common antibiotic resistance gene among the tested isolates. All isolates showing phenotypic resistance to cefoxitin carried the *mecA* gene. Allaion et al. [177] evaluated 136 *S. aureus* isolates from Minas artisanal cheeses, and at least one antibiotic resistance gene was detected in 83.0% of the isolates. Nearly half (47.1%) carried more than one resistance gene. The most frequently detected resistance genes were *tetK* (54.4%) and *mecA* (52.2%), followed by *aacA-aphD*, which was found in 30.0% of the isolates. Aguiar et al. [178] characterized 57 *S. aureus* isolates from artisanal colonial cheese, with penicillin resistance being the most prevalent (33%), followed by resistance to clindamycin (28%), erythromycin (26%), and tetracycline (23%). The evaluated strains also exhibited inducible resistance to clindamycin, with nine isolates classified as multidrug-resistant (MDR).

Pineda et al. [92] characterized the genomes of 54 *S. aureus* isolated from raw milk artisanal cheese in Brazil, identifying antimicrobial resistance genes with phenotypic confirmation of methicillin and tetracycline resistance. The authors also discovered a rich virulome encoding of iron uptake systems, immune evasion mechanisms, and an extensive arsenal of toxins, along with the capacity to form biofilm. These findings suggest that multiple strains circulating in the cheese-producing region pose a potential health risk.

Fontes et al. [89] isolated 227 CoNS from soft cheese in Brazil, and high percentages of antimicrobial resistance were observed for penicillin (78.5%), oxacillin (76.2%), erythromycin (67.8%), gentamicin (47.2%), clindamycin (35.7%), rifampicin (26.8%), azithromycin (14.7%), tetracycline (14.7%), levofloxacin (14.2%), and sulfamethoxazole-trimethoprim (11.9%). All isolated CoNS were susceptible to vancomycin and linezolid. A multiple antibiotic resistance (MAR) index of >0.2 was observed in 80.6% of the isolates. In addition, 81.5% of the isolates carried the *mecA* gene, and 76.2% of these were phenotypically resistant to oxacillin. Nunes et al. [66] isolated CoNS from Minas Frescal cheese in southeastern Brazil, and all 10 evaluated strains showed multiresistance to antimicrobial agents such as beta-lactams, vancomycin, and linezolid. Klempt et al. [175] evaluated 53 CoNS isolates from different cheeses sold in Germany, some of which exhibited resistance to cefoxitin, penicillin, and tetracycline. In addition, several carried genes encoding antibiotic resistance, such as *mecA*, *mecB*, *mecD*, *blaTEM*, *tetK*, and *tetL*.

The detection of MDR staphylococci, including MRSA and MRCNS, in cheese raises serious concerns regarding public health and food safety. These bacteria not only compromise the efficacy of clinical treatments but also act as reservoirs of transferable resistance determinants. Through horizontal gene transfer, MDR staphylococci can disseminate resistance genes to other bacteria present in the cheese microbiota, such as lactic acid bacteria, or later to commensal and pathogenic microorganisms in the human gut following consumption. This gene exchange within the food matrix or during digestion represents a critical future challenge, as it may contribute to the global expansion of antimicrobial resistance (AMR). From a One Health perspective, the circulation of MDR staphylococci among animals, food, and humans highlights the interconnectedness of human, animal, and environmental health and underscores the need for coordinated surveillance and control strategies across all sectors. Therefore, addressing the occurrence of MDR staphylococci in cheese requires integrated actions, including surveillance of AMR in foods, rational antibiotic use in livestock, and improved hygiene practices along the cheese production chain [89,158,179].

### 4.3. Expression of Enterotoxins Genes

SFP is caused by one or more enterotoxins produced by some species and strains of *Staphylococcus*. Although enterotoxin production is associated with CoPS and thermonuclease-positive *S. aureus* (TPS), some CoNS and species that are thermonuclease-negative (TNS) also produce enterotoxins [46,180]. Within *S. aureus*, the regulation of virulence factors is subject to a complex network that integrates host and environmentally derived signals into a coordinated response [181].

The genome of *S. aureus* harbors numerous toxin-encoding genes, which are primarily located on mobile genetic elements [173,182]. This arrangement results in significant variability in toxin production among different *S. aureus* strains [173,183]. Among the various known or strongly suspected toxins and virulence factors that cause specific diseases or symptoms, staphylococcal superantigens (SAgs), comprising SEs, SEls, and TSST-1, are the most prominent [173,182,183].

SAgs are a group of potent immunostimulatory toxins produced by *S. aureus*. SAgs are characterized as pyrogenic toxin superantigens, with the ability to induce SFP and an infection known as Toxic Shock Syndrome (TSS). SAgs share many structural and functional similarities but have distinct characteristics. They are relatively resistant to heat and to proteolytic gastric enzymes such as pepsin and trypsin, allowing them to pass through the digestive tract and head to the site of action. In SFP, SAgs stimulate the vagus nerve endings in the stomach lining that control the emetic response, causing nausea, cramping, vomiting, and diarrhea, appearing abruptly 2–8 h after ingesting food containing these toxins. TSS is a potentially fatal disease characterized by fever, erythematous rash, hypotension, shock, multiple organ failure, and skin desquamation. This toxin-mediated systemic disease was first observed in non-systemic infections by SE producing *S. aureus*. Subsequently, another *S. aureus* toxin, designated as TSST-1 (formerly SEF), was shown to be associated with TSS in menstruating women and in non-menstrual cases [182].

SAgs are single-chain proteins that interact with variable regions of T-cell receptors (TCR V*α* or TCR V*β*), activating a large number of T-cells. This activation triggers massive proliferation and release of pro-inflammatory cytokines, such as IL-1, IL-2, IL-6, γ-interferon, and TNF, potentially leading to lethal TSS. SAgs can also interact with epithelial cells, promoting transepithelial transport and an inflammatory state. Due to their effects on the immune system and ability to induce SFP and TSS, SAgs are classified as pyrogenic toxin superantigens. Similar to TSST-1, they are super antigenic toxins that activate T-cells in a predominantly nonspecific manner, resulting in an excessive immune response that includes polyclonal T-cell activation and massive cytokine release [3,182,184].

SEs belong to a large family of staphylococcal and streptococcal pyrogenic exotoxins, sharing common phylogenetic relationships, structure, function, and sequence homology. SEs are potent gastrointestinal toxins that cause emesis in a not completely understood manner that involves the induction of histamine release from intestinal mast cells [3,184].

SEs are heat-stable, low-molecular-weight (19,000–29,000 Da), single-chain proteins primarily produced by *S. aureus*, though not exclusively. These toxins belong to a major family of serological types, including SEA to SEE and SEIG to SEIJ. The classical enterotoxins, SEA, SEB, SEC1-3, SED, SEE, and SEH, are the main agents responsible for SFP [9,180,184,185]. These toxins function as SAgs, originally identified due to their emetic activity in SFP. This group includes SEs A, B, C, D, E, G, H, I, R, and T, as well as SElJ to SElX, which do not cause emesis or have not been tested in non-human primates. TSST-1, a pyrogenic exotoxin previously known as SEF, is also part of this SAgs group [23,186]. Although the role of certain SEs, SEls, and TSST-1 in foodborne diseases remains unclear and therefore cannot be ruled out, they share structural and functional similarities and have been associated not only with SFP- and TSS-like syndromes, but also with allergic and autoimmune disorders [9,180,184,185].

**Figure 1:**
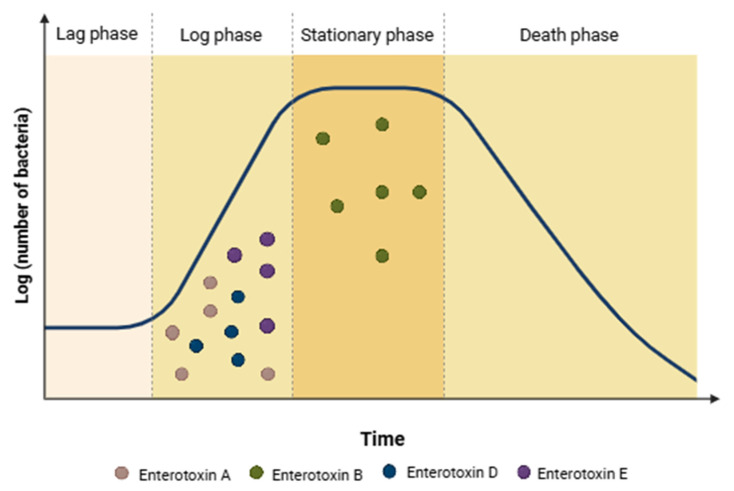
Regulation of enterotoxin production during bacterial growth phases. Based on Derzelle et al. [187].

The expression of genes encoding SEB occurs primarily at the end of the stationary phase, while the production of SEA, SED, and SEE takes place throughout the logarithmic growth phase (Figure 1; [187]). The production of SEA and SEE is not regulated by the accessory gene regulator (*agr*) system [180,184]. In contrast, SEB, SEC, and SED require a functional *agr* system for maximal expression [184]. The *agr* system facilitates cell-to-cell communication through a quorum sensing mechanism, using autoinducing peptides (AIPs) as signaling molecules. Activation of the *agr* system leads to the expression of exotoxins and exoenzymes and is essential for virulence in animal models of skin infection, pneumonia, and endocarditis [181].

The synthesis of SEs depends on temperature, pH, Aw, and the presence/activity of other microorganisms with beneficial or antagonistic interactions. Generally, the production and accumulation of enterotoxins in food occurs when enterotoxigenic staphylococci are capable of proliferating, normally when populations are above 10^5^ CFU/g [180,185,188].

The successful growth of *S. aureus* in diverse environmental conditions is partly due to its ability to express different genes in response to changing conditions. Additionally, *S. aureus* has extraordinary adaptive power to ensure its success as a pathogen [182,189]. This microorganism is capable of detecting different environmental signals and adjusting the production of virulence factors critical for survival in the host, such as cell surface adhesins, extracellular enzymes, and toxins [24,181]. A virulence gene is often susceptible to transcirculatory control by more than one regulatory system and there is cooperation or even competition between these systems to modulate the expression of a given virulence gene [190]. The *agr* quorum sensing mechanism is an important regulatory system of *S. aureus* and contributes to its pathogenicity, playing a key role in the expression of enterotoxins genes [181,190].

SaeRS, a two-component system in *S. aureus* responsible for the production of toxins, immunomodulators, and enzymes, was proven to be essential for virulence in animal models of skin infections and pneumonia [181,191]. The staphylococcal respiratory response regulator (SrrAB) is an oxygen-responsive two-component system that induces the expression of *plc* and *ica*, while repressing *agr*, *tsst-1*, and *spa*. It is essential for defense against neutrophils [181,192]. SrrAB activates *ica* operon transcription and promotes the expression of polysaccharide intercellular adhesin, helping *S. aureus* evade neutrophil-mediated killing during anaerobic growth conditions [192]. ArlRS, another two-component system that regulates autolysis and cell surface properties, promotes MgrA expression while repressing *agr* and autolysis. It is crucial for virulence in animal models of skin infection and endocarditis [193].

SarA is a cytoplasmic regulator that promotes the expression of extracellular proteins and represses *spa*, which encodes staphylococcal protein A. SarA is required for virulence in biofilm infection models [194]. Rot is a cytoplasmic regulator that controls the production of toxins and extracellular proteases in *S. aureus*. Its expression is regulated by the *agr* system, which, when active, prevents *rot* from being translated. Interestingly, in certain conditions where *agr* is inactive (e.g., *agr*-null mutants), mutations in *rot* can restore the virulence of the bacteria. This was demonstrated in a rabbit model of endocarditis [195].

The transition of *S. aureus* from a commensal organism to a pathogen is strongly influenced by host-derived environmental signals, as described by Choueiry et al. [196]. In this study, significantly lower growth of the MRSA strain was observed under aerobic conditions, suggesting that these bacteria were subjected to oxidative stress, which impaired growth. Furthermore, supplementation of culture media with energy substrates and addition of carbon sources facilitated the ability of *S. aureus* to overcome environmental stress and grow, demonstrating a more robustly adaptive metabolism. These authors also noted that changes in growth environments may drive the regulation of virulence in *S. aureus* with the associations of changes in their metabolism with its virulence. Increased expression of the virulence factors *agr-I*, *sea*, *seb*, and *eta* was apparent in the supplemented *S. aureus* cultures [196].

Signal transduction systems that sense cell density, energy levels, and external stimuli facilitate *S. aureus*’s remarkable adaptability to diverse environmental conditions [189,194]. These host environmental signals are crucial in promoting *S. aureus* colonization, allowing bacteria to adapt to different conditions and potentially switch to a pathogenic state when conditions are favorable. Understanding these host–pathogen interactions is critical for managing *S. aureus* infections in clinical settings and understanding enterotoxin expression in food [197].

Current knowledge regarding the regulation of enterotoxin production by *S. aureus* remains limited. It is known that this bacterium can respond to environmental changes through mechanisms involving at least 16 two-component systems, including one dependent on quorum sensing communication, as well as numerous post-translational protein regulators [23,188]. These systems enable the bacterium to rapidly adapt to stress factors by modulating the expression of genes associated with key physiological traits, including enterotoxin production [23,186]. Each system may directly or indirectly control the transcription of specific gene sets, and a single gene may be influenced by multiple systems, resulting in multilayered regulation [186].

During cheese production, various environmental parameters act as signals that modulate the complex regulatory networks of *S. aureus*, directly influencing the expression of virulence genes, including those responsible for enterotoxin synthesis. The lowered pH, resulting from the metabolic activity of lactic acid bacteria, activates two-component systems such as *saeRS* and *agr*, which are sensitive to changes in acidity and bacterial population density (quorum sensing), thereby modulating toxin expression depending on the microenvironment [198,199]. The reduced Aw, typical of aged cheeses, limits the availability of free water, imposing osmotic stress that activates regulators like *sigB*, which mediate stress responses and may suppress virulence gene expression. High salt concentrations, common in cheeses such as Parmesan and Feta, also exert osmotic pressure and can interfere with signaling pathways involving systems like *kdpDE* and *arlRS*, affecting the transcription of genes related to survival and toxin production [61,180]. Additionally, the low temperatures used during cheese ripening (typically between 4 and 12 °C) slow bacterial metabolism and may reduce the activity of temperature-sensitive regulatory systems such as *rot* and *sarA*, which influence enterotoxin expression. Collectively, these parameters create a hostile environment that generally inhibits the growth of *S. aureus* and the production of enterotoxins, especially when effective starter cultures are employed [200]. However, failures in controlling these factors may allow the activation of regulatory pathways that promote toxin expression, posing a risk to food safety [199].

## 5. *Staphylococcal* Food Poisoning (SFP) from Cheese Consumption

SFP occurs due to the consumption of food containing preformed SEs, typically produced when *S. aureus* reaches concentrations of 10^6^ CFU/g or mL in the food matrix [201]. Although Bastos et al. [201] cite 100 ng as a general threshold dose to cause illness, other studies suggest that, for example, even 20–100 ng of SEA may be sufficient [202]. However, the dose response depends on the individual’s sensitivity, body weight, and the specific SE involved [202,203,204,205].

Documented SFP outbreaks associated with cheese consumption, categorized by country and year of occurrence, are summarized in Table 4. No further outbreaks were identified in the literature.

Regarding SFP outbreaks linked to cheeses, [210] reported that four individuals from the same family became ill after consuming fresh Minas cheese, in Brazil. The cheese contained high counts of *S. aureus* (9.3 × 10^7^ CFU/g), and the strains were capable of producing SEA, SEB, SED, and SEE, which were likely responsible for the outbreak, with the main symptoms being nausea, vomiting, diarrhea, and abdominal pain, with no hospitalizations. The average incubation period was approximately one hour.

Pereira et al. [211] reported an outbreak that occurred in 1995 due to consumption of cheese produced in the Minas Gerais state, Brazil. A family of seven individuals consumed the cheese and began to present symptoms of vomiting and diarrhea approximately 4 h later. Analysis of the cheese revealed a high population of *S. aureus* (2.9 × 10^8^ CFU/g) and the presence of SEH. There were no hospitalizations or deaths. In 1999, two additional outbreaks involving *Staphylococcus* and cheeses occurred in Minas Gerais state, Brazil, affecting around 700 people. One outbreak was linked to the consumption of Minas cheese and the other to raw milk. In the first outbreak, analysis of the cheese revealed *S. aureus* levels ranging from 2.4 × 10^3^ to 2.0 × 10^8^ CFU/g, with the production of SEA, SEB, and SEC. In the second outbreak, raw milk samples contained CoNS at counts exceeding 2.0 × 10^8^ CFU/g, along with the production of SEC and SED [212].

From 2014 to 2023, 6874 foodborne disease outbreaks were reported in Brazil, leading to 110,614 illnesses and 12,346 hospitalizations and *S. aureus* was the second leading etiological agent, responsible for 9.7% of cases [217]. In these outbreaks dairy products were responsible for 6.7% of the total number of outbreaks. Unfortunately, no data is available on enterotoxins for these samples.

In Canada, in 1980, 62 individuals presented symptoms of nausea, vomiting, abdominal cramps, and diarrhea after consuming curd cheese, which was present in both boxed lunches and cheeses purchased at retail stores in cities near Montreal. The curd cheese was mainly produced in a cheese factory and distributed to retail stores for preparation of boxed lunches. When analyzed, the curd cheeses contained between 2.0 and 8.0 × 10^7^ *S. aureus*/g, in addition to SEA and SEC. No deaths were reported [206]. In the United Kingdom, in 1981, a family of four consumed Halloumi cheese in brine imported from Cyprus. This cheese, traditionally made with goat’s and sheep’s milk, may also include cow’s milk in some cases. After consumption, all family members developed symptoms typical of SFP. Although *S. aureus* was not isolated, SEA was detected in both the cheese and the brine [209]. Between December 1984 and January 1985, cheese made from raw ewe’s milk at a dairy farm was linked to three outbreaks involving 27 people in the United Kingdom. The people who got ill had severe symptoms, such as violent vomiting and severe diarrhea. SEA was detected in the cheese, although *S. aureus* was not identified. Subsequent testing of milk samples from the dairy revealed the presence of a SEA-producing strain [207].

Kérouanton et al. [208] investigated outbreaks associated with *S. aureus* in France, reporting that between 1981 and 2002, there were 13 incidents involving cheese. The analyzed matrices included raw milk semi-hard cheeses, raw milk soft cheeses, soft cheeses, raw milk cheeses, and sheep’s milk cheeses (raw and pasteurized). The enterotoxins detected in the cheeses were SEA, SEB, and SED. *S. aureus* populations ranged from 1.0 × 10^4^ to 3 × 10^8^ CFU/g, depending on the outbreak and cheese type. Reported symptoms among patients included nausea, vomiting, abdominal cramps, and diarrhea, with no deaths. Ostyn et al. [213] reported six outbreaks occurred in France between October and November 2009 due to SEE in soft cheeses, resulting in 23 cases. The people got ill after consuming soft cheese from one producer with 1.5 × 10^5^ CFU/g and the only type of SE detected in all food samples was SEE (between 0.36 to more than 1.14 ng/g of cheese).

Filipello et al. [215] reported an outbreak in Lombardy, Italy, in 2018, caused by the consumption of artisanal Alm cheese containing SED. This outbreak involved three patients, and all individuals presented abdominal cramps, vomiting, and diarrhea.

Between 2009 and 2016, Cardamone et al. [214] reported four outbreaks in the Sicily region of Italy, linked to the consumption of Primosale cheese, a fresh, soft cheese traditionally made from raw sheep’s milk and produced in Central and Southern Italy. The outbreaks affected a total of 14 individuals, who exhibited similar symptoms in each case, including vomiting, abdominal cramps with or without nausea, and diarrhea. *S. aureus* population ranged from 1.5 × 10^5^ CFU/g in the 2016 outbreak to 4.8 × 10^8^ CFU/g in the 2013 outbreak. SEC was detected in all cheeses associated with the outbreaks, with concentrations exceeding 19 ng/g in the 2011 outbreak and reaching 399.96 ng/g in the cheese from the 2013 outbreak.

In Northern Italy, in 2022, a family of eight reported gastrointestinal symptoms such as vomiting and diarrhea, as well as headaches, after eating sandwiches at a small local restaurant in the Alps region of Piedmont. Food safety agency inspectors collected samples of ham and cheese made with raw milk that the family members had consumed and found CoPS varying between 1.3 × 10^3^ and 8.1 × 10^3^ CFU/g in the cheeses, in addition to SEA to SEE, with SED estimated at 0.649 ng/g. There were no deaths [216].

In Switzerland, in 2007, five individuals presented nausea, abdominal cramps, vomiting, and diarrhea after ingestion of a fresh goat milk cheese, Rabiola. The samples presented counts of CoPS between 6.7 × 10^6^ and 2.6 × 10^7^ CFU/g. The strains were positive for genes (*seg*, *sei*, *sem*, *sen*, and *seo*; [202]). Also in Switzerland, another outbreak affected 14 people, including children and adults, after consuming soft cheese produced from raw cow milk, Tomme cheese. The soft cheese contained SEA (>6 ng/g) and SED (>200 ng/g). Counts of 10^7^ CFU/g of CoPS were detected. No deaths were reported [202].

According to the European Food Safety Authority (EFSA) and European Centre for Disease Prevention and Control (ECDC), in 2022, *S. aureus* toxins were responsible for 137 outbreaks (0.02% of the total of 5763 reported cases), resulting in 148 hospitalizations and 4 deaths [218]. Between 2007 and 2018, 8730 foodborne disease outbreaks caused by seven pathogens were reported in Japan. Among these, *S. aureus* was responsible for 448 outbreaks, none of which resulted in fatalities. Additionally, 2.6% of outbreaks linked to dairy product consumption were attributed to *S. aureus* [219].

The analysis of the outbreaks compiled in Table 4 shows that the classical enterotoxins SEA, SEB, SEC, and SED were the most frequently implicated in cheese-related SFP cases, reaffirming their central role in staphylococcal foodborne diseases. Soft and fresh cheeses made from raw or insufficiently pasteurized milk, especially those derived from sheep or cow’s milk, appear to be most frequently involved, likely because these products are more susceptible to contamination and provide favorable conditions for *S. aureus* growth when temperature control is inadequate. In several outbreaks, bacterial counts exceeded 10^5^–10^6^ CFU/g, supporting the hypothesis that temperature abuse during manufacturing, storage, or distribution, combined with high initial contamination levels, constitutes one of the main contributing factors.

Abiotic factors such as temperature, pH, Aw, redox potential, NaCl concentration and oxygen availability, in addition to bacterial antagonism, influence the growth and enterotoxin production by *S. aureus* in food [220,221]. These factors may help explain the relatively low number of *S. aureus* outbreaks associated with cheese. The optimal temperature range for both growth and enterotoxin production by *S. aureus* is 34–40 °C. The optimal pH for growth is 6 to 7, while for enterotoxin production it is 7 to 8. The ideal Aw for both growth and enterotoxin production is 0.99, although reports indicate enterotoxin production can occur between 0.86 and 0.99 [220]. Moreover, Schelin et al. [220] reported that the presence of lactic acid bacteria in cheese, such as *Lactococcus lactis*, can inhibit the transcription of genes responsible for enterotoxin production, such as *sec*, *selk*, *seg*, and *seh*. These characteristics suggest that although cheeses may provide conditions that support growth and toxin production, it does not offer the ideal environment which may partially explain the limited number of confirmed SFP outbreaks in the scientific literature.

Furthermore, the number of cases may also be underestimated, as SFPs are frequently underreported: symptoms are typically mild and self-limiting, leading affected individuals not to seek medical attention. In addition, the lack of laboratory confirmation of enterotoxins and the limited capacity of surveillance systems, especially in developing regions, further hinder the determination of the true prevalence of staphylococcal intoxications. Therefore, although the intrinsic characteristics of the cheese matrix may restrict enterotoxin production, the existing data likely represent an underestimation of reality, emphasizing the need to strengthen diagnostic capacity and ensure continuous monitoring of dairy products within food safety programs.

## 6. Control of *Staphylococcus* in Cheeses

Controlling pathogens like enterotoxin-producing *S. aureus* is essential to ensure the safety of cheeses. At the farm level, maintaining animal health and adopting hygienic milking practices are critical to minimizing microbiological contamination of raw milk [222]. Preventing mastitis and implementing good agricultural practices significantly reduces the risk of contamination with spoilage and pathogenic bacteria, including those from the *Staphylococcus* group [91]. In cheese production facilities, strict hygiene protocols, good manufacturing practices (GMPs), proper equipment design and maintenance, adequate production flow, as well as monitoring of production surfaces, raw materials and final products are important barriers to reducing cross-contamination with harmful bacteria. Additionally, the adoption of a hazard analysis and critical control points (HACCP) plan and a proactive food safety culture can minimize contamination, ensure product safety and promote a better working environment [223]. Controlled ripening conditions, such as proper temperature and pH, also help prevent *S. aureus* proliferation, while regular health checks for food handlers mitigate risks of contamination during handling.

Further down the supply chain, appropriate storage conditions at retail are important to hinder bacterial growth. Preventing cross-contamination during slicing and repackaging ensures that cheese remains safe for consumers. At home, proper handling, hygiene, and storage practices are key to reducing contamination and microbial growth, spoilage and to keep the product safe [224]. A comprehensive approach, from the farm to table, is necessary to effectively control *S. aureus* and other foodborne pathogens and safeguard public health.

### Emerging Control Strategies

Several innovative approaches have been proposed to complement conventional control measures against *S. aureus* and its enterotoxins in cheeses. Beyond traditional cleaning-in-place (CIP) systems using peracetic acid or sodium hypochlorite, novel biological and technological solutions have gained attention. For instance, lytic bacteriophages specifically targeting *S. aureus* have shown potential for reducing contamination in milk and cheese matrices [225]. Similarly, the use of competitive probiotic strains, such as certain *Lactobacillus* and *Enterococcus* species, can inhibit pathogen colonization and toxin gene expression [226]. Advances in nanotechnology have also introduced innovative tools, including functionalized magnetic microrobots capable of selectively removing *S. aureus* cells from milk without disrupting beneficial microbiota [227]. Furthermore, advances in rapid detection techniques, including real-time PCR, chromogenic media, and biosensors, have enhanced the early identification of enterotoxigenic strains, improving traceability and enabling more timely interventions [228]. While several of these innovations are still undergoing validation, their integration into the existing framework of GMP presents a promising avenue for strengthening microbial safety in both artisanal and industrial cheese production.

## 7. Microbiological Criteria for *Staphylococcus* and Enterotoxins in Cheeses

Microbiological criteria for CoPS, including *S. aureus* and their SEs in cheeses, are determined by regulatory bodies and differ among countries. In Brazil, the National Agency for Sanitary Vigilance (ANVISA) aligns its regulations with international standards, particularly those from Codex Alimentarius, focusing on controlling both CoPS and SEs in dairy products (Table 5). Detection of SEs in cheeses triggers corrective actions, including product destruction and potential product recalls.

In the European Union, Commission Regulation (EC) No 2073/2005, amended by EC No 1441/2007, specifies that the presence enterotoxins in cheese used as raw material should be monitored, especially when levels of CoPS exceed 10^5^ CFU/g of product [233]. This threshold is considered critical as it marks the potential for enterotoxin production, which can lead to foodborne illness outbreaks. The EFSA’s guidelines for the detection of SEs focus on preventing contamination, particularly in raw milk cheeses, which are more susceptible to staphylococcal contamination during the production and ripening stages.

The Food and Drug Administration (FDA), through its Bacteriological Analytical Manual (BAM), provides specific methods for detecting SEs in foods, including cheese. While it does not set explicit limits for CoPS in cheeses, it emphasizes the importance of testing for enterotoxins, given their heat stability, which allows them to withstand pasteurization processes that eliminate the bacteria themselves [234]. The United States Department of Agriculture (USDA) recommendation is similar to the FDA.

These regulations aim to reduce the risk of SFP through a combination of good hygiene practices, appropriate processing conditions, and regular testing at various stages of cheese production, storage, and distribution. However, beyond their practical application, it is essential to critically assess the scientific rationale and limitations underlying these microbiological criteria. The thresholds established by different international regulations reflect not only epidemiological evidence but also cultural and technological factors. For example, the European Union allows higher limits for raw milk cheeses, considering the protective role of the competitive microbiota and the preservation of traditional sensory characteristics [218]. Nevertheless, this approach raises concerns about the uniformity of consumer protection, as outbreaks have been reported even in products that meet these microbiological standards.

A major limitation of these criteria is the weak correlation between CoPS or *S. aureus* counts and the actual presence of enterotoxins in cheese. Preformed enterotoxins are thermostable and can persist when CoPS or *S. aureus* populations are reduced by pasteurization or microbial competition [89,158,179]. Furthermore, focusing exclusively on CoPS and *S. aureus* may underestimate risk, since several CoNS and CoVS species also harbor enterotoxin genes and can express them under favorable environmental conditions. Thus, assessing food safety based solely on CoPS or *S. aureus* enumeration may not accurately reflect the real potential for enterotoxins contamination.

In this context, integrating enterotoxin quantification with *Staphylococcus* spp. enumeration, including CoPS, CoNS, and CoVS, offers a more comprehensive approach to evaluating contamination risk, even though it would implicate more financial costs. However, significant analytical challenges remain, as available detection and quantification methods vary in sensitivity and typically target only a limited subset of enterotoxins. Moreover, environmental and storage conditions, such as temperature and duration, can influence in situ toxin synthesis, meaning that bacteria may produce enterotoxins over time even if none are initially detected. In summary, these considerations highlight the need for harmonized microbiological criteria that take into account both *Staphylococcus* counts and enterotoxin detection and quantification, thereby improving the reliability of food safety assessments and ensuring more consistent consumer protection.

## 8. Conclusions and Perspectives

As advocated in this review, the genera *Staphylococcus* exhibits a dual role in cheese production, where certain CoNS can be beneficial by contributing to ripening and enhancing flavor and texture, while pathogenic strains, especially *S. aureus* and other CoPS, pose food-safety risks due to SE production. The persistence of antibiotic-resistant strains, including MRSA, further complicates risk management, and at the farm level both CoPS and CoNS are relevant because of their capacity to cause disease in the herd (e.g., mastitis). Given that low concentrations of SE can cause food poisoning, strict control measures are required. Additionally, it is necessary to advance research on enterotoxin regulatory mechanisms under production conditions, to implement stringent hygiene and GMP protocols, and to maintain rigorous temperature control throughout the chain, while monitoring raw materials and livestock health to reduce mastitis risk.

To render these recommendations actionable for producers, a practical approach is advised. In daily routine, producers should adopt basic, low-cost measures, reinforce personal and equipment hygiene, rapid cooling of milk (even simple cooling baths where refrigeration is limited), routine mastitis screening (visual inspection and California mastitis test—CMT) with segregation of milk from suspicious animals, and preference for well-characterized commercial starters rather than uncontrolled environmental CoNS strains. At an intermediate level, periodic outsourcing of bulk-tank testing to regional laboratories, culture or quantitative polymerase chain reaction (qPCR) for *S. aureus*, targeted enterotoxin assays via partner labs when sensory changes or incidents occur, and short worker-training sessions on GMPs may yield large safety gains at moderate cost. Advanced interventions, including molecular screening, are valuable but are best implemented through university or cooperative partnerships rather than as daily practice.

Adoption of this coordinated, evidence-based strategy enables the intentional use of beneficial CoNS to improve cheese quality while minimizing the risk posed by pathogenic *Staphylococcus* spp., antibiotic-resistant strains, and enterotoxin production. Ongoing research and periodic review of practices remain essential to preserve both product quality and food safety.

Although remarkable progress has been made in understanding *Staphylococcus* ecology and control in cheeses, important questions remain open. There is a need for real-time molecular or biosensor-based methods capable of detecting enterotoxigenic strains directly in dairy matrices, allowing preventive or immediate corrective actions. Research on bacteriophage therapy, natural antimicrobials, and microbial interactions in complex cheese ecosystems could offer new and sustainable control options, especially for artisanal production. Understanding how climate, terroir, and seasonal variations influence the composition and behavior of staphylococcal communities also deserves greater attention, as these factors shape both the beneficial and pathogenic members of the microbiota. Finally, global regulatory frameworks should evolve toward harmonized, risk-based microbiological criteria that reflect modern insights into microbial ecology and production diversity. Addressing these gaps will be key to ensuring that cheese making continues to combine tradition, innovation, and safety in a fast changing world.

## Figures and Tables

**Table 1:** Species of the coagulase-positive (CoPS), coagulase-negative (CoNS), and coagulase-variable (CoVS) staphylococci.

Coagulase-Positive Staphylococci (CoPS) Species		
*Staphylococcus argenteus*	*Staphylococcus cornubiensis*	*Staphylococcus lutrae*
*Staphylococcus aureus*	*Staphylococcus delphini*	*Staphylococcus pseudintermedius*
*Staphylococcus coagulans*	*Staphylococcus intermedius*	*Staphylococcus schweitzeri*
Coagulase-negative staphylococci (CoNS) species		
*Staphylococcus americanisciuri*	*Staphylococcus edaphicus*	*Staphylococcus pettenkoferi*
*Staphylococcus argensis*	*Staphylococcus epidermidis*	*Staphylococcus piscifermentans*
*Staphylococcus arlettae*	*Staphylococcus equorum*	*Staphylococcus pragensis*
*Staphylococcus auricularis*	*Staphylococcus felis*	*Staphylococcus pseudolugdunensis*
*Staphylococcus borealis*	*Staphylococcus gallinarum*	*Staphylococcus pseudoxylosus*
*Staphylococcus brunensis*	*Staphylococcus haemolyticus*	*Staphylococcus ratti*
*Staphylococcus caeli*	*Staphylococcus hominis*	*Staphylococcus rostri*
*Staphylococcus caledonicus*	*Staphylococcus hsinchuensis*	*Staphylococcus saccharolyticus*
*Staphylococcus canis*	*Staphylococcus kloosii*	*Staphylococcus saprophyticus*
*Staphylococcus capitis*	*Staphylococcus leei*	*Staphylococcus schleiferi*
*Staphylococcus caprae*	*Staphylococcus lloydii*	*Staphylococcus shinii*
*Staphylococcus carnosus*	*Staphylococcus lugdunensis*	*Staphylococcus simiae*
*Staphylococcus casei*	*Staphylococcus lyticans*	*Staphylococcus simulans*
*Staphylococcus chromogenes*	*Staphylococcus marylandisciuri*	*Staphylococcus succinus*
*Staphylococcus cohnii*	*Staphylococcus massiliensis*	*Staphylococcus taiwanensis*
*Staphylococcus condimenti*	*Staphylococcus microti*	*Staphylococcus ureilyticus*
*Staphylococcus croceilyticus*	*Staphylococcus muscae*	*Staphylococcus warneri*
*Staphylococcus debuckii*	*Staphylococcus nepalensis*	*Staphylococcus xylosus*
*Staphylococcus devriesei*	*Staphylococcus pasteuri*	-
*Staphylococcus durrellii*	*Staphylococcus petrasii*	-
Coagulase-variable staphylococci (CoVS) species		
*Staphylococcus agnetis*	*Staphylococcus roterodami*	-
*Staphylococcus hyicus*	*Staphylococcus singaporensis*	-

Based on NCBI [7]; Casanova et al. [10]; Velázquez-Guadarrama et al. [17]; Foronda-García-Hidalgo [18]; and Madhaiyan et al. [19].

**Table 2:** Characterization of genetic element, activities, and source of staphylococcal enterotoxins (SEs), SEs-like (SEls), and toxic shock syndrome toxin-1 (TSST-1).

Name	Gene	Genetic Element	Size (kDa)	Crystal Structure Solved *	Stability	Superantigenic Activity	Emetic Activity	Source
*Staphylococcal* enterotoxins (SEs)								
SEA	*sea*	Prophage	27.1	Yes	Heat- and protease- stable	Yes	Yes	Food poisoning, dairy products, human, bovine, caprine, ovine
SEB	*seb*	SaPI3, chromosome, plasmid	28.4	Yes	Heat-stable	Yes	Yes	Food poisoning, dairy products, human, bovine, caprine, ovine
SEC	*sec*	SaPI	27.5–27.6	Yes	Heat-stable	Yes	Yes	Food poisoning, dairy products, human, bovine, caprine, ovine
SEC-1	*sec*	SaPI	27.5–27.6	Nd	Presumed heat-stable	Yes	Yes	Human
SEC-2	*sec*	SaPI	27.5–27.6	Yes	Heat-stable	Yes	Nd	Human
SEC-3	*sec*	SaPI	27.5–27.6	Yes	Heat-stable	Yes	Yes	Human
SED	*sed*	Plasmid	26.9	Yes	Heat-stable	Yes	Yes	Food poisoning, bovine
SEE	*see*	Prophage (hypothetical location)	26.4	No	Heat-stable	Yes	Yes	Food poisoning, unpasteurized milk soft cheese
SEG	*seg*	*egc1*, *egc2*, *egc3*, *egc4*	27.0	Yes	Heat-stable	Yes	Yes	Bovine
SEH	*seh*	Transposon	25.1	Yes	Heat-stable	Yes	Yes	Empyema human
SEI	*sei*	*egc1*, *egc2*, *egc3*	24.9	Yes	Heat-stable	Yes	Yes	Mastitis cows, humans
SEs-like (SEls)								
SElJ	*selj*	Plasmid	28.5	No	Nd	Yes	Nd	Epidemiologically implicated in food poisoning
SElK	*selk*	SaPI1, SaPI3, SaPI5, SaPIbov1, prophages	26.0	Yes	Nd	Yes	Nd	Human
SElL	*sell*	SaPIn1, SaPIm1, SaPImw2, SaPIbov1	26.0	No	Nd	Yes	Yes	Human
SElM	*selm*	*egc1*, *egc2*	24.8	No	Nd	Yes	Yes	Bovine
SElN	*seln*	*egc1*, *egc2*, *egc3*, *egc4*	26.1	No	Nd	Yes	Yes	Human
SElO	*selo*	*egc1*, *egc2*, *egc3*, *egc4*, transposon	26.7	No	Nd	Yes	Yes	Human
SElP	*selp*	Prophage	27.0	No	Nd	Yes	Yes	Human, ulcer
SElQ	*selq*	SaPI1, SaPI3, SaPI5, prophage	25.0	No	Nd	Yes	Yes	Human
SElR	*selr*	Plasmid	27.0	No	Nd	Yes	Yes	Human
SElS	*sels*	Plasmid	26.2	No	Nd	Yes	Yes	Not found
SElT	*selt*	Plasmid	22.6	No	Nd	Yes	Yes	Not found
SElU	*selu*	*egc2*, *egc3*	27.1	No	Nd	Yes	Nd	Human
SElV	*selv*	*egc4*	Nd	No	Nd	Yes	Nd	Not found
SElW	*selw*	*egc4*	Nd	No	Nd	Yes	Nd	Human
SElX	*selx*	Chromosome	Nd	No	Nd	Yes	Nd	Milk, raw meat, human
SElY	*sely*	Chromosome	Nd	No	Nd	Yes	Nd	Human
SElZ	*selz*	Chromosome	Nd	No	Nd	Nd	Nd	Bovine
Toxic shock syndrome toxin-1 (TSST-1)								
TSST-1	*tst/TssT*	Chromosome	Nd	Nd	Heat- and protease-stable	Yes	No	Human

* The structures of some enterotoxins can be found at http://www.ebi.ac.uk/ebisearch/search.ebi?db=macromolecularStructures&t=%22staphylococcal+enterotoxin%22&requestFrom=navigateYouResults (accessed on 31 October 2025). SaPI = *Staphylococcus aureus* Pathogenicity Island; Nd = not determined; *egc* = enterotoxin gene cluster. Based on Cieza et al. [61]; Etter [62]; Fernández et al. [63]; and Berry et al. [64].

**Table 3:** Resistance genes to different antibiotic classes and their encoded proteins in *Staphylococcus* spp.

Main Target	Antibiotic Class	Resistance Gene	Encoded Protein
Cell wall synthesis	Beta-lactam	*mecA*	Penicillin-binding protein 2a (PBP2a)
		*mecA1*	
		*blaZ*	Beta-lactamase
		*blaTEM*	
	Glycopeptide	*bleO*	Bleomycin resistant proteins
		*vanA*	Vancomycin/teicoplanin A-type resistance protein
	Phosphonic acid	*fosB-saur*	Metallothiol transferase
Folic acid synthesis	Diaminopyrimidine	*dfrG*	Dihydrofolate reductase
	Trimethoprim	*dfrA12*	Dihydrofolate reductase
		*dfr17*	
Nucleic acid synthesis	Quinolone	*gyrA*	DNA gyrase subunit A
Nucleic acid and protein synthesis	Nucleoside	*sat-4*	Streptothricin N-acetyltransferase and streptothricin
Protein synthesis	Aminoglycoside	*aacA-aphD*	6′-aminoglycoside N-acetyltransferase/2″-aminoglycoside phosphotransferase
		*aadA2*	Spectinomycin 9-adenylyltransferase
		*aadA5*	Aminoglycoside-3′-adenylyltransferase
		*ant(4′)-Ia*	Aminoglycoside adenyltransferase
		*aph(2″)-Ih*	Aminoglycoside 2″-phosphotransferase
		*aph(3′)-IIIa*	Aminoglycoside 3′-phosphotransferase
	Fusidane	*fusB*	2-domain zinc-binding protein
		*fusC*	
	Lincosamide	*lnuA*	Lincosamide nucleotidyltransferase
	Lincosamide/ macrolide/streptogramin	*ermC*	rRNA adenine N-6-methyltransferase
	Lincosamide/pleuromutilin/streptogramin/	*salA*	Iron-sulfur cluster carrier protein
		*vgaA-lc*	ABC transporter
	Macrolide	*mphC*	Macrolide 2′-phosphotransferase
	Macrolide/streptogramin	*msrA*	Peptide methionine sulfoxide reductase
	Phenicol	*fexA*	Chloramphenicol/florfenicol exporter
		*cmlA1*	Bcr/CflA family efflux transporter
	Tetracycline	*tetK*	Tetracycline resistance protein
		*tetL*	
		*tet38*	Tetracycline efflux MFS transporter

Based on Alkuraythi et al. [171].

**Table 4:** Occurrence of staphylococcal food poisoning (SFP) associated with cheese consumption in different locations.

Year	Location	Product	Enterotoxin Type	Symptoms	Number of Patients (Deaths)	Reference
1980	Canada	Curd cheese	SEA, SEC	Nausea, vomiting, abdominal cramps, and diarrhea	62 (0)	[206]
1981	United Kingdom	Halloumi cheese	SEA	Nausea, vomiting, abdominal cramps, and diarrhea	4 (0)	[207]
1981	France	Raw milk semi-hard cheese	SEA	Unknown	4 (0)	[208]
1983	France	Raw milk semi-hard cheese	SEA, SED	Vomiting and abdominal cramps	20 (0)	[208]
1983	France	Raw milk soft cheese	Absent	Vomiting and diarrhea	4 (0)	[208]
1985	France	Soft cheese	SEB	Vomiting and diarrhea	2 (0)	[208]
1985	France	Soft cheese	SEB	Vomiting and diarrhea	3 (0)	[208]
1985	United Kingdom	Raw ewe’s milk cheese	SEA	Nausea, vomiting, abdominal cramps, and diarrhea	27 (0)	[209]
1986	France	Sheep’s milk cheese	SEB	Unknown	Unknown	[208]
1988	Brazil	Fresh Minas cheese	SEA, SEB, SED, SEE	Nausea, vomiting, abdominal cramps, and diarrhea	4 (0)	[210]
1995	Brazil	Minas cheese	SEH	Vomiting and diarrhea	7 (0)	[211]
1997	France	Raw milk cheese	Present but not specified	Unknown	43 (0)	[208]
1998	France	Raw milk cheese	Present but not specified	Vomiting, abdominal cramps, and diarrhea	47 (0)	[208]
1998	France	Raw milk semi-hard cheese	Absent	Vomiting and abdominal cramps	10 (0)	[208]
1999	Brazil	Minas cheese	SEA, SEB, SEC	Vomiting, dizziness, chills, headaches, and diarrhea	378 (0)	[212]
2000	France	Raw sheep’s milk cheese	SEA	Unknown	Unknown	[208]
2001	France	Sliced soft cheese	SEA	Nausea, vomiting, abdominal cramps, and diarrhea	2 (0)	[208]
2001	France	Raw milk semi-hard cheese	SED	Vomiting	17 (0)	[208]
2002	France	Raw sheep’s milk cheese	SEA	Nausea, vomiting, abdominal cramps, and diarrhea	43 (0)	[208]
2007	Switzerland	Robiola cheese	SEG, SEI, SEM, SEN, SEO	Nausea, vomiting, abdominal cramps, and diarrhea (in some cases)	5 (0)	[202]
2009	France	Soft cheese	SEE	Nausea, vomiting, abdominal cramps, diarrhea, and fever (in some cases)	23 (0)	[213]
2009	Italy	Soft raw sheep milk cheese	SEC	Vomiting and abdominal cramps	2 (0)	[214]
2011	Italy	Soft raw sheep milk cheese	SEC	Nausea, vomiting, abdominal cramps, and diarrhea	3 (0)	[214]
2013	Italy	Soft raw sheep milk cheese	SEC	Vomiting, abdominal cramps, and diarrhea	6 (0)	[214]
2014	Switzerland	Tomme cheese	SEA, SED	Vomiting, abdominal cramps, severe diarrhea, and fever	14 (0)	[202]
2016	Italy	Soft raw sheep milk cheese	SEC	Vomiting, abdominal cramps, and diarrhea	6 (0)	[214]
2018	Italy	Alm cheese	SED	Vomiting, abdominal cramps, and diarrhea	3 (0)	[215]
2022	Italy	Raw milk cheese	SEA, SEB, SEC, SED, SEE	Vomiting, diarrhea, and headaches	8 (0)	[216]

SE, followed by a letter, denotes the identified *Staphylococcus* enterotoxin (SE), for example: SEA, SEB, SEC, SED, SEE, SEG, SEH, SEI, SEM, SEN, and SEO.

**Table 5:** Microbiological criteria for coagulase-positive staphylococci (CoPS), *Staphylococcus aureus*, and *Staphylococcus* enterotoxins (SEs) in different countries or regions of the world.

Country or Region	Microbiological Criteria						
	CoPS, *S. aureus*, or SEs	n	c	m	M	Notes	Reference
Australia	CoPS	5	2	100	1000	All types of cheese	[229]
Brazil	CoPS	5	2	100	1000	All types of cheese	[230]
	SEs	5	0	absence	-	All types of cheese	
Canada	*S. aureus*	5	2	1000	10,000	Cheese made from an unpasteurized source	[231]
China	*S. aureus*	5	2	100	1000	All types of cheese	[232]
European Union	CoPS	5	2	10,000	100,000	Cheese made from raw milk	[233]
		5	2	100	1000	Cheese made from mild heat treated milk	
		5	2	10	100	Unriped soft cheese made with pasteurized milk	
United States	*S. aureus*	-	-	-	10,000	All dairy products	[234]
	SEs	-	-	not detected	-	All dairy products	

CoPS = coagulase-positive staphylococci; *S. aureus* = *Staphylococcus aureus*; SEs = *Staphylococcus* enterotoxins; n = the number of sample units that are to be independently and randomly drawn from a lot; c = the maximum allowable number of sample units yielding unsatisfactory results (in two-class plans) or with marginally acceptable quality (in three-class plans); m = the microbiological limit that separates acceptable samples from unacceptable ones (in two-class plans) or from marginally acceptable quality (in three-class plans); M = the second microbiological limit that separates acceptable samples from unacceptable ones (in three-class plans).

## Data Availability

No new data were created or analyzed in this study. Data sharing is not applicable to this article.

## Abbreviations

The following abbreviations are used in this manuscript:

Aap	Accumulation-associated protein
AMR	Antimicrobial resistance
ANVISA	National Agency for Sanitary Vigilance
ATP	Adenosine triphosphate
AIP	Autoinducing peptide
Aw	Water activity
BAM	Bacteriological Analytical Manual
Bap	Biofilm-associated protein
CMT	California mastitis test
CIP	Cleaning-in-place
ClfA/ClfB	Clumping factors
CoNS	Coagulase-negative staphylococci
CoPS	Coagulase-positive staphylococci
CoVS	Coagulase-variable staphylococci
ECDC	European Centre for Disease Prevention and Control
EFSA	European Food Safety Authority
ELISA	Enzyme-linked immunosorbent assay
EPS	Extracellular polymeric substance
ET	Exfoliative toxin
EU/EEA	European Union/European Economic Area
FDA	Food and Drug Administration
Fib	Fibrinogen-binding protein
FnBP	Fibronectin-binding protein
GISA	Intermediate-susceptible *Staphylococcus aureus* to glycopeptides
GI	Genomic island
GMP	Good manufacturing practices
GRAS	Generally recognized as safe
HACCP	Hazard analysis and critical control points
LA-MRSA	Livestock-associated methicillin-resistant *Staphylococcus aureus*
MAR	Multiple antibiotic resistance
MDR	Multidrug-resistant
MRCNS	Methicillin-resistant coagulase-negative staphylococci
MRSA	Methicillin-resistant *Staphylococcus aureus*
MRSP	Methicillin-resistant strains
MSSA	Methicillin-susceptible *Staphylococcus aureus*
MSCRAMMs	Microbial surface components recognizing adhesive matrix molecules
NaCl	Sodium chloride
PIA	Polysaccharide intercellular adhesion
PCR	Polymerase chain reaction
PNAG	Poly-N-acetylglucosamine
Pls	Plasmin-sensitive protein
PSM	Phenol-soluble modulin
PVL	Panton-Valentine leukocidin
qPCR	Quantitative polymerase chain reaction
SasC/SasG	*Staphylococcus aureus* surface proteins
SCCmec	*Staphylococcal* chromosomal cassette mec
SE	*Staphylococcal* enterotoxin
SEl	*Staphylococcal* enterotoxin-like protein
SFP	*Staphylococcal* food poisoning
SrrAB	*Staphylococcal* respiratory response regulator
SSSS	*Staphylococcal* scalded skin
